# Selection and validation of miR-1280 as a suitable endogenous normalizer for qRT-PCR Analysis of serum microRNA expression in Hepatocellular Carcinoma

**DOI:** 10.1038/s41598-020-59682-0

**Published:** 2020-02-21

**Authors:** Muhammad Yogi Pratama, Luisa Cavalletto, Claudio Tiribelli, Liliana Chemello, Devis Pascut

**Affiliations:** 1 Fondazione Italiana Fegato - ONLUS, Liver Research Center, AREA Science Park, Basovizza, Trieste Italy; 2 Department of Medicine – DIMED, University-Hospital of Padova, Padova, Italy; 3 Universitas Hasanuddin, Faculty of Medicine, Makassar, Indonesia

**Keywords:** Biological techniques, Cancer, Molecular biology

## Abstract

Normalization procedures for the qRT-PCR analysis of miRNA in biological samples are recommended to reduce the variability caused by pre-analytical factors. Since there is no universal standardized normalization strategy for miRNA qRT-PCR studies, we conducted a throughout study to evaluate a panel of small non-coding RNAs (sncRNAs) as reference gene candidate for biomarker studies in serum samples of patients with hepatocellular carcinoma (HCC). Five sncRNAs (miR-1280, miR-1275, SNORD-116, SNORD-68, and U6) were chosen as candidate of reference genes. This study included 122 patients with HCC and was organized into a “pilot phase” consisting of 20 serum samples of HCC patients, and a “validation phase” of 102 patients. Expression level of these candidates were analyzed by qRT-PCR. Assessment of gene stability was performed using four different integrative platforms (geNorm NormFinder, Bestkeeper, and the Delta Ct method). To determine the gene stability during the follow-up of the patient, we extend the analysis of the validation cohort at T1 (1 month after treatment) and T2 (6 month after treatment). MiR-1280 was identified as the most stably expressed reference gene in both pilot and validation phase also during the follow-up. MiR-1280 appears a reliable reference gene candidate in biomarker studies.

## Introduction

Hepatocellular carcinoma (HCC) is one of the most common types of malignancies worldwide^1^. It is primarily known as the fourth-most common cause of mortality from cancer, contributing to 781.631 death in 2018^1^. Late diagnosis and the limited therapeutic approaches represent the ongoing challenges in liver oncology, responsible for the poor survival in patients^2–4^. Currently, numerous translational studies have been focused on the identification of circulatory biomarkers related to early diagnosis and prognosis^5,6^.

Since their discovery in 1993, microRNAs (miRNAs) have been considered promising non-invasive biomarkers with wide application in many clinical settings^7,8^. MiRNAs are fundamental regulators of many cellular processes^9,10^ and their alteration contributes to the disease onset, maintenance, and progression^11^. It has been demonstrated that miRNAs present in a stable cell-free form in the blood are not affected by temperatures, pH, technical handling, and storages. Different miRNAs profiles in blood circulation have been linked to cancer, including HCC, emphasizing the broad potential of miRNAs as a diagnostic and prognostic biomarkers^12–14^.

One of the most popular technique to study the expression of miRNome in a biological sample is the quantitative reverse transcription polymerase chain reaction (qRT-PCR), which is widely preferred due to significant advantages such as sensitivity, large dynamic range, accuracy, and the convenient requirement of starting materials^15,16^. Standardization and normalization procedures are sometimes recommended for the comparison of miRNA expression between different samples to reduce the variability caused by pre-analytical factors such as specimen collection, processing, and storage^17–20^. Currently, there is no universal standardized normalization strategy for miRNA qRT-PCR studies. Ideally, suitable endogenous normalizers should be selected according to the experimental conditions, considering disease type, population, and type of biological material. Most of the studies used various normalization procedures or even disregarded the use of any reference gene^21–23^. Several most common intracellular housekeeping genes (HK) for qRT-PCR such as GADPH, 18S rRNA, HPRT, and Actin are no longer utilizable in biofluid profiling experiments. The ideal endogenous control should be expressed at a constant level in all samples and exhibit storage stability, extraction efficiency equivalent to that of the target gene, ideally belonging to the same RNA class^24,25^.

The small nuclear RNA U6 is frequently used as normalizer in miRNA profiling studies, including in HCC^26,27^. However, various reports showed of its instability across different sample sets^26,28–30^. Studies have suggested several stable expressed miRNAs as endogenous controls however performing diversely in different biological sources, strengthening the need of proper reference genes for biomarker studies in serum samples^21,31^.

Hence, we conducted a two-phase study to evaluate a panel of small non-coding RNAs (sncRNAs) as reference gene candidate for biomarker studies from 223 serum samples of HCC patients. We first explored the expression stability of 5 HK candidates in a pilot phase. The results were subsequently, confirmed through a validation phase in a larger set of patients. In addition, their stability was tested during the follow-up of the patients.

## Results

### The expression of reference gene candidates in the pilot cohort

**Figure 1:**
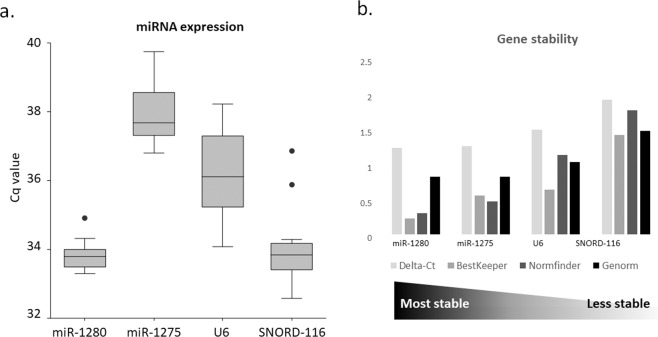
(**a**) Average sncRNAs expression in the pilot cohort. (**b**) Overall sncRNAs stability calculated with Delta-Ct method, BestKeeper, Normfinder and GeNorm algorithms during the pilot phase. Candidates genes are ranked based on their overall stability.

Two commonly used reference genes, U6 and SNORD-68^27,28,32^ were selected as normalizers for the discovery of circulating biomarkers. We also included some other sncRNAs that have never been tested before as candidate biomarkers, such as miR-1275, miR-1280 and SNORD116. To evaluate the stability of the five candidates, we assessed their expression by qRT-PCR in the pilot cohort of 20 serum samples of HCC patients. The average Cq value of the candidates ranged from 33.79 to 37.98. MiR-1275 and U6 sncRNA had the highest raw Cq expression among all samples (Fig. 1a, Table S1), while miR-1280 and SNORD-116 are the most abundant sncRNAs among the samples (Fig. 1a). In our experimental setting, SNORD-68 was only detectable in few samples and therefore was excluded from the subsequent analyses.

The stability of the candidate sncRNAs was further analyzed by using the four analysis tools described in the Materials and Methods section. The rank of gene stability between the candidates is reported in Table 1 and Fig. 1b. MiR-1280 was considered the most stable gene among all samples by all the statistical algorithms used, while SNORD-116 was calculated as the least stable gene. GeNorm and BestKeeper suggested some thresholds for considering candidates genes as stable genes. Based on the study of Vandesompele *et al*., the recommended threshold for stable genes have a GeNorm M value lower than 1.5, while the M value for the highly stable gene is below 0.5^33^. Meanwhile, the stability threshold for Bestkeeper is SD lower than 1.00. MiR-1280, miR-1275 and U6 are considered stable genes both from GeNorm and from BestKeeper, with both respectively having M value lower than 1.5 and a SD value lower than 1.00. SNORD-116 overcomes the BestKeeper cut-off; however, it has M value of 1.53 in GeNorm. Hence, to clarify the stability of SNORD-116, we decided to include it for the next study phase together with the other candidates. It worth to be noticed that in this pilot phase, none of the candidates is considered highly stable based on the GeNorm algorithm.

**Table 1:** The cumulative ranking and gene stability values of reference candidate genes in the pilot cohort using four different integrative platforms consist of GeNorm, NormFinder, BestKeeper, and Delta Ct.

candidate	Pilot Cohort (20 cases)				
	rank	GeNorm (M value)	NormFinder (SV value)	BestKeeper (SD value)	Delta Ct (SD value)
miR-1280	1	0.88	0.36	0.29	1.29
miR-1275	2	0.88	0.52	0.61	1.31
U6	3	1.09	1.18	0.69	1.55
SNORD-116	4	1.53	1.82	1.47	1.97

### The expression of reference gene candidates in the validation cohort

To validate the results obtained from the pilot phase, we extended the analysis to the validation cohort consisting of 102 subjects with HCC. In the validation cohort, we observed a different mean Cq for miR-1275 and SNORD-116 (Table S2). The expression of miR-1275 and SNORD-116 was lower compared to the pilot cohort, with most of the samples having a Cq greater than 40. The low expression near to the detection limit determines the high variance values (Table S2).

**Figure 2:**
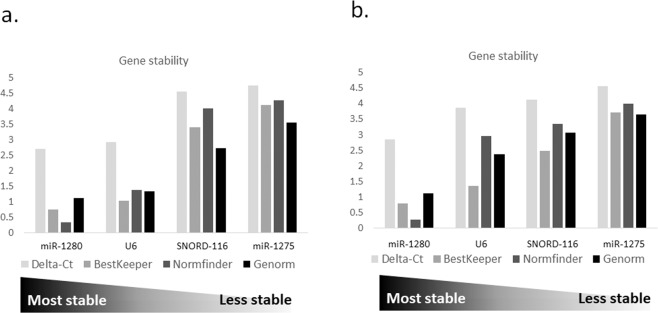
(**a**) Overall sncRNAs stability calculated with Delta-Ct method, BestKeeper, Normfinder and GeNorm algorithms in the validation cohort. Candidates genes are ranked based on their overall stability. (**b**) Overall sncRNAs stability calculated with Delta-Ct method, BestKeeper, Normfinder and GeNorm algorithms in the validation cohort. The overall gene stability was calculated for all the three time points considered in the study (T0, T1 and T2). Candidates genes are ranked based on their overall stability.

When analyzing the stability of the candidate sncRNAs in the validation cohort, miR-1280 was confirmed as the most stable candidate, ranking first also in this group of patients, while miR-1275 and SNORD-116 were classified as the most unstable genes (Fig. 2a). In the validation cohort, the overall ranking changed significantly (Table 2) and miR-1280 was the only candidate with a BestKeeper SD value lower than 1.0. While considering the GeNorm M value, miR-1280 and U6 are the only candidate with stability below the threshold value (Table 2).

**Table 2:** The cumulative ranking and gene stability values of reference candidate genes in the validation cohort.

candidate	Validation Cohort (102 cases)				
	rank	GeNorm (M value)	NormFinder (SV value)	BestKeeper (SD value)	Delta Ct (SD value)
miR-1280	1	1.13	0.34	0.76	2.71
U6	2	1.35	1.39	1.03	2.93
SNORD-116	3	2.74	4.01	3.40	4.56
miR-1275	4	3.55	4.28	4.12	4.76

### Reference genes stability during patient’s follow-up

We assessed the intra-patient variability by extending the analysis during the patients’ follow-up, at two different time points. Out of the 102 samples analyzed at a baseline (T0, at HCC diagnosis and before the initiation of HCC treatment), 64 patients were analyzed also at T1 (one month after treatment), and 37 at T2 (six months after treatment). The overall stability among all samples is reported in Fig. 2b and Table 3. The gene stability calculated with every single algorithm did not differ from the overall stability calculated at T0 alone for all the candidates, except for U6, in which the M and SV values doubled when considering all the time points (Fig. 2a,b and Tables 2 and 3). MiR-1280 perfomed as the best candidate for all the considered algorithms, being the only one below the GeNorm and BestKeeper threshold (Table 3). The stability of miR-1280 was also analyzed according to the different clinical characteristics of patients, and found to be not statistically significant for all the considered variables, thus strengthening the potential of miR-1280 as a normalizer (Table S4).

**Table 3:** Overall ranking and gene stability values of reference candidate genes calculated for all the three time points.

candidate	Validation Cohort				
	rank	GeNorm (M value)	NormFinder (SV value)	BestKeeper (SD value)	Delta Ct (SD value)
miR-1280	1	1.12	0.27	0.79	2.86
U6	2	2.37	2.95	1.36	3.87
SNORD-116	3	3.06	3.36	2.49	4.13
miR-1275	4	3.66	4.00	3.37	4.56

### The effect of different normalizers to the expression of a target miRNA

In order to determine the effect of different normalizers on the expression of circulating miRNAs, we profiled miR-1246, miR-4454 and miR-4443 during the follow-up of the patients. The three miRNAs were selected according to their possible relevance in HCC^34,35^. We compared the performances of the two highest-ranked normalizers, miR-1280 and U6. The normalized averaged mean differences among the three-time point are reported in Table 4. The differential expression of miR-1246, miR-4454 and miR-4443 resulted statistically significant when normalized with miR-1280 points (p = 0.007, p = 0.038, p = 0.04, respectively) (Table 4), while no statistical significance was obtained when using U6 as normalizer.

**Table 4:** Changes in the average miR-1246, miR-4454 and miR-4443 expression according to the different normalizer.

miRNA *vs* normalizer	Mean expression (±SD)			
	T0	T1	T2	p value
**miR-1246** ***vs*** **miR-1280**	0.89 ± 0.16	0.83 ± 0.14	0.86 ± 0.09	0.007
**mir-1246** ***vs*** **U6**	0.84 ± 0.16	0.82 ± 0.14	0.82 ± 0.18	0.25
**miR-4454** ***vs*** **miR-1280**	0.96 ± 0.11	0.94 ± 0.09	0.90 ± 0.08	0.038
**mir-4454** ***vs*** **U6**	0.91 ± 0.11	0.93 ± 0.11	0.88 ± 0.098	0.17
**miR-4443** ***vs*** **miR-1280**	1.18 ± 0.10	1.15 ± 0.10	1.14 ± 0.07	0.04
**mir-4443** ***vs*** **U6**	1.13 ± 0.10	1.14 ± 0.10	1.12 ± 0.10	0.68

## Discussion

The potential use of miRNAs as a non-invasive circulating biomarker has gained much attention in the past couple of years. Currently, there is a large amount of data available regarding miRNAs in various type of cancer diseases, including HCC. However, there are inconsistencies and high variability of data in each study group, making difficult to compare the results from one to another cohort^36,37^. This leads to the lack of reproducibility of results, that limit the potential use of miRNAs in the real clinical practice. One of the possible issue might be the lack of a standardized method to measure the expression levels of miRNA. qRT-PCR is considered the most popular method for miRNA analysis. This method relies on the selection of reference or housekeeping genes to normalize the expression data and prevent an intra or inter heterogeneity between samples. Currently, there is no consensus in the use of endogenous reference genes. The different experimental settings and methods, as well as the heterogeneity of the population and the disease hamper the identification of suitable reference genes for circulating miRNA studies in HCC. In this study, we evaluated five reference genes candidates in 223 serum samples from two different cohorts of patients with HCC. To the best of our knowledge, this is the first study also aimed to define genes stability during the HCC patient’s follow-up, thus focused on identifying  reliable housekeeping miRNAs to be used in HCC studies.

Previous studies, frequently used U6 sncRNA in miRNAs analysis profile, particularly in HCC^26,27^. However, various reports showed its instability across different sample sets^26,28–30^. Several studies reported contradictory results about the comparison with an other miRNA as a reference gene for the normalization of the circulating miRNA expression in cancer setting^38–40^. For example, miR-16 has been described both as a suitable or unsuitable normalizer in serum samples of breast cancer patients^38–40^. Therefore, we believed it is important to identify a stable candidate as a reference gene in order to prevent a contradictory result.

In the present study, five small circulating RNAs were evaluated as candidate reference genes for biomarker profiling analysis in HCC. The analysis performed during the pilot phase ranked the candidate genes based on their average stability calculated through four different algorithms. SNORD-68 was discarded from this preliminary analysis due to its low expression in our setting in contrast with previous data where this sncRNA has been used as an endogenous normalizer for miRNA serum profiling in liver diseases^41^. This difference might be due to the amount of starting RNA used in our experimental setup, as described also in studies from *Motawi and coll*. (2016) that used lower quantities of RNA samples^41,42^. Gene stability analysis ranked miR-1280 as the most stable gene, even if, it did not reach the 0.5 cut-off required for a “highly stable gene” according to GeNorm. Despite the contrasting information presented in literature about the use of U6 as normalizer^26,30^, we considered U6 as a possible housekeeping candidate in this initial evaluation. U6 has been proposed and widely used as normalizer in many miRNA studies.

When we validated the results in a larger cohort of HCC serum samples, we confirmed the potential of miR-1280 as a reference gene. SNORD-116 and miR-1275 were poorly expressed in the validation cohort with high variance among samples, ranking third and fourth, respectively. This result underlines some diversities between the cohorts that can justify the selection of different normalizers. Nevertheless, miR-1280 was considered as a good normalized in both pilot and validation cohort, suggesting a possible application in several other studies regarding HCC. An important and novel aspect of this study is that we evaluated the stability of candidate genes during patient follow-up at three different time points T0, T1 and T2.

The overall gene rank stability calculated for all the samples (T0 + T1 + T2) is consistent with the stability previously calculated at T0 alone, but less consistent with the results obtained in the pilot cohort. This evidenced the low intra-patient variability compared to the inter-patient variability, even across time. Interestingly, the stability of U6 was decreased when analyzed at all the time points, underlining its unreliability as normalizers for time series analysis.

MiR-1280 and miR-1275 performed best in the pilot cohort. However, in the validation cohort that considers larger set of patients, miR-1280 resulted the best candidate, while miR-1275 ranked as the most unstable gene. This discrepancy might be due to the unclear role of miR-1275 in the disease. One study reported miR-1275 as differently expressed in HCC *vs* cirrhotic and healthy controls, and being responsible of the *in vitro* regulation of tumor invasion process^43^.

In order to determine the effect of different combinations of reference genes in the normalization of a miRNA of interest, the RT-qPCR expression profile of three miRNAs related to HCC^34,35^, miR-1246, miR4454 and miR4443, was assessed at all time points. The differential expression of the three miRNAs resulted statistically significant only when normalized with miR-1280 compared to U6, which ranked second after the overall stability evaluation. These results underline that the selection of the most suitable miRNA normalizers allows the detection of subtle differences among samples. Hence, miRNA expression stability must be carefully assessed in each specific experimental setting to determine the most suitable reference gene.

Among the candidates tested, miR-1280 showed the lowest intra- and inter- variability. Despite the limitations of the present study, consisting in the small sample size of the pilot cohort and the use a single qRT-PCR methodology based on SYBR green, we identified miR-1280 as a reliable reference miRNA, that can be used as normalizer in serum biomarkers studies in HCC.

## Materials and Methods

### Study design

To identify the most suitable endogenous control for miRNA biomarker studies in HCC we organize the study as follow:

1. *Pilot phase*. The expression of five sncRNAs candidates was assessed in 20 serum samples. The Gene stability was determined by GeNorm, NormFinder, BestKeeper and Delta-Ct method. Four out of the five candidates were further tested in the validation cohort (Fig. 3).

**Figure 3:**
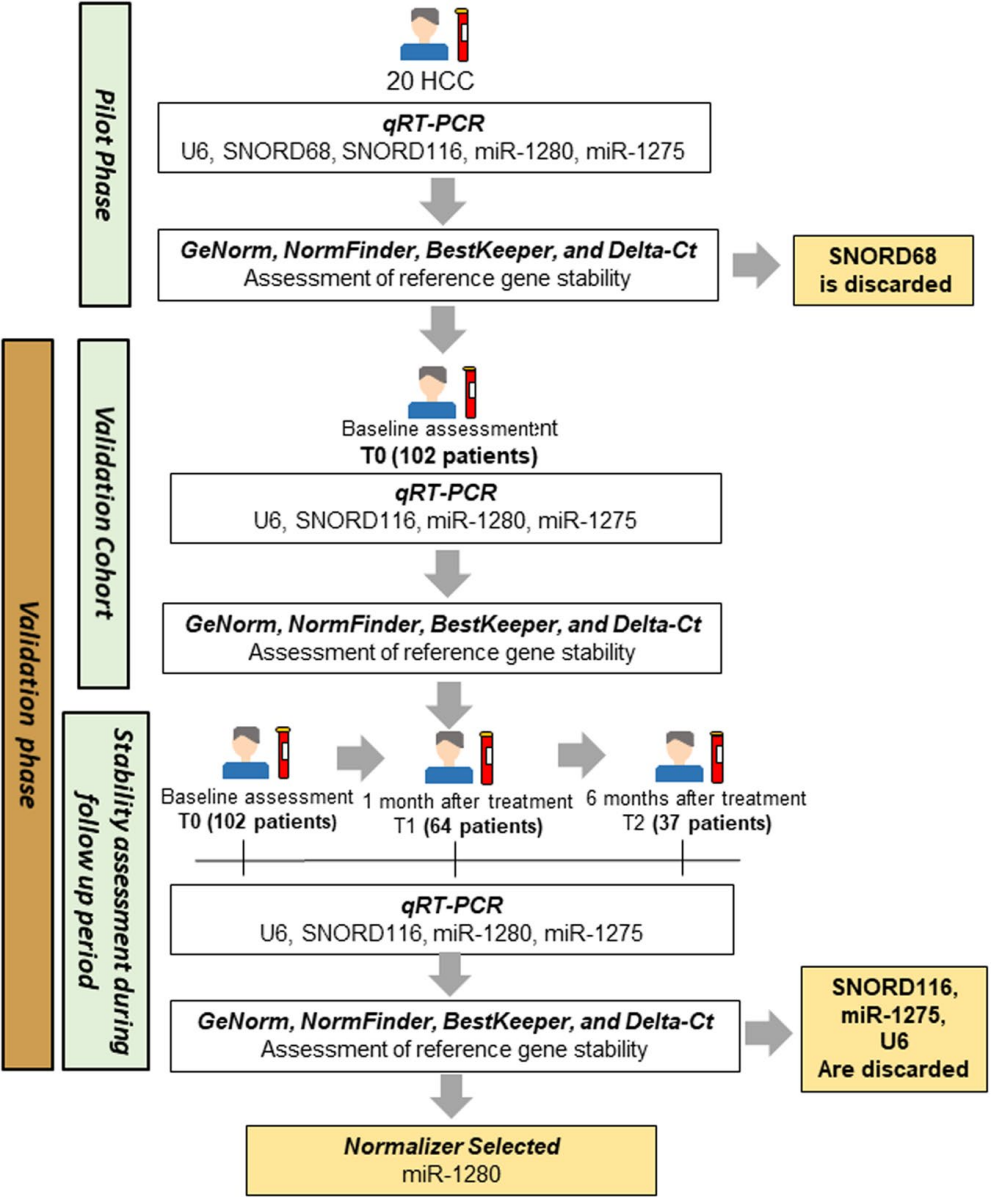
Workflow of the study.
2. *Validation phase*. SncRNAs candidates were tested by qRT-PCR in an independent cohort of 102 patients at baseline. To determine the gene stability during follow-up, the analysis was extended T1 (1 month after treatment) and T2 (6 month after treatment). After gene stability evaluation a miRNA candidate was selected as endogenous normalizer for circulating miRNA biomarker studies in HCC (Fig. 3)

### Patients’ characteristics

Serum samples from 122 HCC patients were obtained from two different centers. The Pilot cohort consists of 20 HCC patients from the Hospital of Padova (Padova, Italy). The validation cohort consists of 102 HCC patients from the Cattinara Hospital (Trieste, Italy). Samples were collected at the time of HCC diagnosis before any treatment. Informed consent was provided, and the study was approved by the Institutional Review Board of the local ethic committee and conducted according to the rules of the Helsinki declaration. Clinical and demographic features of patients are described in Table S4.

In the pilot cohort, recruited cases showed a mean age of 59 years (49–68 95%CI), with gender male in 65% and mean BMI of 25,8 + 4,1 (+SD). The majority of cases were defined with a CPT A (65%), while all had viral etiology. The HCC score system (BCLC), subtyped cases in 4 groups (O/A/B/C-D), respectively 4/3/10/3 cases, with a very early (0 stage) or early tumor (stage A) burden in 35%. All but 2 (90%) showed AFP <20 ng/ml.

In the validation cohort participants were predominantly male (79%) with a mean age of 70 (48–87 95%CI). The etiology of chronic liver disease was alcohol abuse and/or metabolic in the majority of HCC patients, while the viral component was present in 45% of the patient either alone or in combination with alcohol abuse and/or metabolic syndrome. The great majority of the patients were CTP score A (74.5%).

HCC was staged according to the BCLC staging system into 4 groups, BCLC 0, A, B, C/D respectively in the 8%, 61%, 25%, and 6% of the patients. As for AFP level, 69% had lower than 20 ng/mL, while 13% showed AFP level above 400 ng/mL.

### Serum collection and RNA Isolation

Serum samples were obtained from 10 mL of whole blood collected in sterilized tubes and centrifuged at 3000 rpm/minute in a refrigerated centrifuge. Supernatants were transferred in 1 mL Eppendorf tubes and subsequently froze at −80 °C for long-term storage. SncRNAs were isolated from 300 ul of serum using the Nucleospin miRNA Plasma Isolation Kits – Biofluids (Macherey-Nagel, Germany). MicroRNAs were quantified in a Qubit 2.0 Fluorometer (Thermo Fischer Scientific, Waltham, MA USA) by using the Qubit microRNA Assay Kit (Thermo Fischer Scientific, Waltham, MA USA) following the manufacturer instructions.

### qRT-PCR

Thirty nanograms of sncRNAs were reverse transcribed by using the qScript microRNA cDNA Synthesis Kit (Quantbio, Beverly, MA) according to manufacturer instruction. The RT-qPCR was performed with the PerfeCTa SYBR Green SuperMix (Quantbio, Beverly, MA) in a CFX-96 thermal cycler (Bio-Rad Laboratories, Hercules CA) according to manufacturer instructions. All reactions were run in duplicate in a 25 uL reaction. Cq values >45 were considered as negative, and the melting point curves were observed for all assays to verify primer specificity. Primers for miR-1275, miR-1280, miR-1246 and SNORD68 were purchased from Sigma-Aldrich (Merck KGaA, Darmstadt, Germany) while the sequences for primers of U6, U6 (5′-CGCTTCGGCAGCACATATAC-3′) and SNORD116 (5′-TGATGATTCCCAGTCAAACATTC-3′) were ordered from Metabion International AG (steinkirchen Germany).

### Assessment of reference gene stability

We evaluated the stability of the candidates small RNAs using the four integrative platforms, geNorm NormFinder, Bestkeeper, and the Delta Ct method. The GeNorm algorithm calculates the average expression stability value (M) defined as the average pairwise variation in a particular gene with all other potential reference genes in a multi-steps procedure by performing a removal of reference gene with the lowest stability (M) and repeatedly calculating the remaining candidate until deciding one most stable reference gene^44^. NormFinder uses an ANOVA-based model to consider intra- and inter-group variation to evaluate the expression stability of the candidate gene. The stability value (SV) ranks the set of candidate normalization genes according to their expression stability in a given sample set^45^. BestKeeper generates an index of stability based on the geometric mean of Cq values of candidate genes followed by a pairwise Pearson correlation analysis to rank each of the candidates in the index^46^. The comparative delta-CT method calculates the stability of each gene by obtaining the standard deviation of Cq differences for each pair of reference gene candidates^47^.

### Evaluation of the normalization effect

To determine the effect of the endogenous reference gene selected in this study, the expression levels of miR-1246 were measured and normalized by using miR-1280 and U6 as reference genes candidates. Relative expression levels were reported as 2^−ΔCt^. The Kruskal-Wallis test in One Way ANOVA procedure was used to determine statistically significant differences among the samples. Statistical analysis was performed by using NCSS 11 Software (2016) (NCSS, LLC. Kaysville, Utah, USA, ncss.com/software/ncss). P-values of <0.05 were considered statistically significant.

### Ethics approval and consent to participate

All the patients provided written informed consent and patient anonymity has been preserved. Investigation was conducted according to the principles expressed in the Declaration of Helsinki. The study was approved by the local Ethic committee Prot N 3386/AO/14, approved on 12/01/2015 and Cod. NRC AOP0357, being part of a Regional Survey Program within HUB centers on antiviral therapy for chronic hepatitis and cirrhosis associated to HCV infection and by the regional Ethical Committee (full name: Comitato Etico Unico Regionale FVG C.E.U.R, No. 14/2012 ASUITS).

## Supplementary information

Supplementary information.
