# Supplementary material for: Selection and validation of miR-1280 as a suitable endogenous normalizer for qRT-PCR Analysis of serum microRNA expression in Hepatocellular Carcinoma
Source: Sci Rep. 2020 Feb 21;10:3128. doi: 10.1038/s41598-020-59682-0 (PMC7035418; doi:10.1038/s41598-020-59682-0)
Supplement: Supplementary file 1 — Supplementary information. [file 41598_2020_59682_MOESM1_ESM.docx]

**Selection and validation of miR-1280 as a suitable endogenous normalizers for qRT-PCR Analysis of serum microRNA expression in Hepatocellular Carcinoma**

**Muhammad Yogi Pratama^1,3,#^,** Cavalletto Luisa^2,#^, Tiribelli Claudio^1^, Lilliana Chemello^2,^***** and Pascut Devis^1^,

1. Fondazione Italiana Fegato - ONLUS, Liver Research Center, AREA Science Park, Basovizza, Trieste, Italy.
2. Department of Medicine – DIMED, University-Hospital of Padova, Italy.
3. Universitas Hasanuddin, Faculty of Medicine, Makassar, Indonesia.

**Keywords:** Normalizers, endogenous controls, house keeping genes, qRT-PCR, HCC, serum miRNAs, small RNAs, circulatory miRNAs.

**# These Authors share common authorship**

*** Corresponding author**

**Lilliana Chemello**

Department of Medicine - DIMED

University-Hospital of Padova

Via Giustiniani, 2, 35128 - Padova, Italy

e-mail [liliana.chemello@unipd.it](mailto:liliana.chemello@unipd.it)

**SUPPLEMENTARY TABLES**

**Table S1.** The descriptive statistics of raw Cq expression among the reference genes candidate in the pilot phase.

| **sncRNA** | **Mean Cq (95%CI)** | **Variance (95%CI)** |
| --- | --- | --- |
| **miR-1280** | 33.79 (33.61-33.97) | 0.14 (0.08-0.31) |
| **miR-1275** | 37.98 (37.59-38.37) | 0.70 (0.40-1.48) |
| **U6** | 36.28 (35.64-36.92) | 1.51 (0.84-3.50) |
| **SNORD-116** | 33.99 (33.39-34.59) | 1.16 (0.62-2.88) |

**Table S2.** The descriptive statistics of raw Cq expression among the reference genes candidate in the validation phase.

| **sncRNA** | **Mean Cq (95%CI)** | **Variance (95%CI)** |
| --- | --- | --- |
| **miR-1280** | 33.33 (33.11-33.55) | 1.18 (0.91-0.1.60) |
| **miR-1275** | 40.89 (40.03-41.77) | 19.24 (14.80-26.06) |
| **U6** | 34.97 (34.69-35.25) | 1.99 (1.53-2.70) |
| **SNORD-116** | 43.23 (42.38-44.07) | 17.38 (13.37-23.53) |

**Table S3.** Demographic characteristics

|  | **pilot Cohort (n=20)** | **Validation Cohort (n=102)** | |
| --- | --- | --- | --- |
| **Patients characteristics** |  | |  |
| Age (mean, 95%CI) | 59 (49-68) | | 70 (48-87) |
| Sex (M/F), n° | 13/7 | | 81/ 21 |
| **Etiology, n°** |  | |  |
| Alcohol metabolic | 0 | | 56 |
| Alcohol metabolic viral | 4 | | 10 |
| Viral | 16 | | 36 |
| **Disease scores, n°** |  | |  |
| CTP A/B/C | 13/7/0 | | 73/22/3 |
| BCLC 0/A/B/C-D | 4/3/10/3 | | 8/62/26/6 |
| **Alpha fetoprotein, n°** |  | |  |
| <20 ng/mL | 18 | | 54 |
| 20 - 400 ng/mL | 2 | | 14 |
| >400 ng/mL | 0 | | 10 |

**Table S4.** The ΔCq expression of miR-1280 according to each clinical variables at each time point (validation phase).

| **Clinical Characteristics** |  |  | **ΔCq MiR-1280 expression** | | | | | |  |
| --- | --- | --- | --- | --- | --- | --- | --- | --- | --- |
|  |  | n | T0 (mean ± SD) | P value | T1 (mean ± SD) | P value | T2 (mean ± SD) | P value |  |
| **Sex** | Male | 78 | 0.90 ± 0.63 | 0.92 (ns) | 0.08 ± 0.14 | 0.51 (ns) | 0.048 ± 0.03 | 0.12 (ns) |  |
|  | Female | 20 | 0.91 ± 1.06 |  | 0.05 ± 0.052 |  | 0.07 ± 0.061 |  |  |
| **Etiology** | Viral | 54 | 0.074 ± 0.06 | 0.53 (ns) | 0.052 ± 0.06 | 0.16 (ns) | 0.05 ± 0.02 | 0.07 (ns) |  |
|  | Metabolic | 21 | 0.093 ± 0.06 |  | 0.07 ± 0.07 |  | 0.081 ± 0.06 |  |  |
|  | Viral-Metabolic | 16 | 0.06 ± 0.04 |  | 0.161 ± 0.31 |  | 0.03 ± 0.015 |  |  |
| **BCLC** | 0 | 6 | 0.09 ± 0.06 | 0.22 (ns) | 0.075 ± 0.082 | 0.99 (ns) | 0.053 ± 0.04 | 0.45 (ns) |  |
|  | A | 57 | 0.07 ± 0.06 |  | 0.071 ± 0.15 |  | 0.067 ± 0.05 | 0.46 (ns) |  |
|  | B | 25 | 0.10 ± 0.07 |  | 0.073 ± 0.075 |  | 0.06 ± 0.05 |  |  |
|  | C/D | 6 | 0.06 ± 0.02 |  |  |  |  |  |  |
| **Child-Pugh** | A | 67 | 0.073 ± 0.06 | 0.20 (ns) | 0.06 ± 0.065 | 0.40 | 0.06 ± 0.045 | 0.35 (ns) |  |
|  | B | 25 | 0.092 ± 0.07 |  | 0.11 ± 0.24 |  | 0.038 ± 0.012 |  |  |
| **AFP (ng/ul)** | <20 | 50 | 0.076 ± 0.052 | 0.22 (ns) | 0.07 ± 0.15 | 0.89 (ns) | 0.06 ± 0.04 | 0.82 (ns) |  |
|  | 20-400 | 12 | 0.060 ± 0.02 |  | 0.087 ± 0.10 |  | 0.05 ± 0.01 |  |  |
|  | >400 | 10 | 0.108 ± 0.11 |  | 0.093 ± 0.14 |  | 0.044 ± 0.03 |  |  |

Data are expressed as mean ±SD, statistical analysis was performed by using the Kwuskal-Wallis test.
